# Förderung aktiver Mobilität im Alter durch Stadtgestaltung

**DOI:** 10.1007/s00103-024-03922-5

**Published:** 2024-07-10

**Authors:** Sabine Baumgart, Gabriele Bolte

**Affiliations:** 1 Institut für Public Health und Pflegeforschung, Abteilung Sozialepidemiologie, Universität Bremen, Grazer Str. 4, 28359 Bremen, Deutschland; 2 BPW Stadtplanung, Bremen, Deutschland

**Keywords:** Ältere Bevölkerung, Zufußgehen, Fahrradfahren, Teilhabe, Gesundheitliche Chancengerechtigkeit, Older population, Walking, Cycling, Participation, Health equity

## Abstract

Angesichts einer alternden Gesellschaft ist die Erhaltung einer eigenständigen Mobilität bis in das hohe Alter eine wichtige Zielsetzung. Das mentale und körperliche Wohlbefinden hängt nicht nur von dem individuellen Gesundheitsstatus, sondern wesentlich auch von den räumlichen Bedingungen ab. Darauf können Kommunalpolitik und kommunale Verwaltungen Einfluss nehmen, insbesondere die städtebauliche Planung. In diesem Diskussionsbeitrag werden Perspektiven von Public Health und Stadtplanung auf Stadtentwicklung und Mobilität vor dem Hintergrund von gesundheitlicher Chancengerechtigkeit zusammengeführt.

Die Ergebnisse der AFOOT (Alternd zu Fuß oder mit Fahrrad: urban mobil ohne Stress)-Querschnittstudie zu sozialräumlichen Bedingungen in Klein- und Mittelstädten im Nordwesten Deutschlands und dem Zufußgehen und Radfahren von älteren Menschen zeigen die Bedeutung von Wohnumweltfaktoren wie Nähe von Alltagszielen, Fußwege- und Fahrradinfrastruktur sowie Wegeverbindungen. Präferenzen für die Gestaltung einer alternsgerechten Wohnumgebung und Qualitäten des öffentlichen Raums bestehen hinsichtlich städtebaulicher Gestaltqualität, Aufenthaltsqualität und Sicherheit im öffentlichen Raum.

Für eine Verbesserung der räumlichen Gegebenheiten bedarf es einer Erfassung der Situation durch definierte Indikatoren und eines Monitorings sowie der Integration von Perspektiven älterer Menschen. Strategien und Maßnahmen zur Förderung aktiver Mobilität im Alter zielen auf die multifunktionale Gestaltung öffentlicher Räume, die Priorisierung aktiver Mobilität auf Alltagswegen und die Gewährleistung der Erreichbarkeit von Alltagszielen durch Siedlungsentwicklung. Die sektorübergreifende Zusammenarbeit von Stadtplanung, Verkehrsplanung und Public Health ist für die Förderung der aktiven Mobilität und der Gesundheit älterer Menschen essentiell.

## Abstract (en)

In an ageing society, maintaining independent mobility into old age is an important objective. Mental and physical wellbeing depends not only on individual health status, but also to a large extent on the spatial conditions. Local politics and municipal administrations can influence this, especially in urban planning. This discussion article brings together perspectives from public health and urban planning on urban development and mobility against the background of health equity.

The results of the AFOOT (Securing urban mobility of an ageing population) cross-sectional study on socio-spatial conditions in small- and medium-sized towns in northwestern Germany and walking and cycling by older people show the importance of residential environmental factors such as proximity to everyday destinations, walking and cycling infrastructure, and street connectivity. Preferences for the design of an age-friendly living environment and the quality of public spaces exist in terms of urban design quality, quality of stay, and safety in public spaces.

In order to improve spatial conditions, the situation needs to be recorded using defined indicators and monitoring, and the perspectives of older people need to be integrated. Strategies and measures to promote active mobility in old age are aimed at the multifunctional design of public spaces, the prioritization of active mobility on everyday trips, and ensuring the accessibility of everyday destinations through urban development. Cross-sectoral cooperation between urban planning, transport planning, and public health is essential to promote the active mobility and health of older people.

## Einleitung

Angesichts einer alternden Gesellschaft ist die Erhaltung einer eigenständigen Mobilität bis in die letzten Lebensjahre eine wichtige Zielsetzung. Aktive Mobilität mit dem Fahrrad oder zu Fuß wirkt gesundheitserhaltend und -fördernd, sowohl durch die mit diesen Mobilitätsformen verbundene körperliche Aktivität selbst als auch durch die Ermöglichung sozialer Teilhabe und eigenständigen Lebens [1–4]. Gesundes Altern ist als ein Prozess zu verstehen, der von den Wohnbedingungen und Merkmalen der gebauten Wohnumwelt entscheidend beeinflusst wird. So fördern fußgängerfreundliche Nachbarschaften die täglichen Aktivitäten außerhalb der Wohnung und das transportbezogene Zufußgehen von älteren Menschen [5, 6]. Kommunalpolitik und kommunale Verwaltungen, insbesondere die städtebauliche Planung, nehmen Einfluss auf die Verhältnisse in der Wohnumgebung. Dadurch beeinflussen sie auch das Verhalten älterer Menschen und eröffnen ihnen überhaupt erst die Möglichkeiten, trotz altersgemäßer gesundheitlicher Einschränkungen aktiv zu Fuß oder mit Rad mobil zu sein. Somit bestimmen die räumlichen Lebensverhältnisse wesentlich das Mobilitätsverhalten älterer Menschen, die ihrerseits durch die Nutzung des Raums dessen Entwicklung beeinflussen.

Dabei ist es von Bedeutung, ob ältere Menschen in einem großstädtischen urbanen Umfeld mit vielfältigen Versorgungs- und Dienstleistungsangeboten leben oder in einer ländlichen Region in einer Kleinstadt. Es gibt zwar vielfältige Evidenz für die Wirksamkeit von Interventionen in der städtischen Wohnumwelt hinsichtlich der aktiven Mobilität, sie ist jedoch insofern begrenzt, als die meisten Studien in dichtbesiedelten städtischen Wohnumwelten durchgeführt wurden [3, 7].

Hinzu kommen soziale Ungleichheiten bei Umwelt und Gesundheit: In den letzten Jahren hat die Forschung zu Umweltgerechtigkeit vermehrt den Nachweis für soziale Ungleichheiten in den Wohnbedingungen und der gebauten Wohnumwelt erbracht [8]. In Bezug auf eine gesunde und gerechte Stadtplanung schlägt Corburn [9] das Modell einer „adaptive urban health justice“ vor mit Komponenten wie integrierter Entscheidungsfindung und multidimensionalem Monitoring. Erst in wenigen Städten finden sich inzwischen multisektorale Ansätze von räumlicher Planung, Verkehrsplanung und Öffentlichem Gesundheitsdienst, um gemeinsame Lösungsansätze für Probleme im Wohnumfeld der Bevölkerung zu finden und so die Gesundheit nachhaltig zu fördern [10]. In Deutschland fehlt diese systematische Zusammenarbeit des Planungs- und Gesundheitssektors bislang ebenfalls, insbesondere im Bereich der Bewegungsförderung im Alter durch Stadtgestaltung.

In der Lancet-Serie „Urban design, transport and health“ veröffentlichte Überblicksarbeiten [11–13] betonen

- die Rolle und Möglichkeiten der räumlichen Planung, durch die Integration des Themas der aktiven Mobilität in die tägliche Arbeit, die Gesundheit und das Wohlbefinden der Bevölkerung zu verbessern und soziale Ungleichheiten bei Gesundheit zu bekämpfen;
- den Bedarf an lokalen Strategien in der Verwaltung, die das Zufußgehen, Fahrradfahren und den Öffentlichen Nahverkehr priorisieren;
- den Nutzen, durch einen verstärkten Wissenschafts-Praxis-Transfer den Einfluss der gesundheitsbezogenen Forschung auf Entscheidungen der Stadtplanung zu erhöhen.

An diesen Punkten setzte das Projekt AFOOT^1^ an. Es hatte zum Ziel, als Intervention strategische Verbindungen zwischen Stadtplanung und Public Health zu knüpfen, um aktive Alltagsmobilität als eine Form von körperlicher Aktivität im Alter zu fördern. AFOOT verfolgte dabei einen inter- und transdisziplinären Ansatz, um Anknüpfungspunkte für die Abschätzung von Folgen für gesundheitliche Chancengerechtigkeit sowie Teilhabe in räumlichen Planungsprozessen, insbesondere in Klein- und Mittelstädten in der Metropolregion Bremen-Oldenburg, zu identifizieren. AFOOT fokussierte auf bereits bestehende Planungsprozesse und zielte auf Veränderungen in der Planungspraxis, wodurch gesundheitliche Chancengerechtigkeit in der Bevölkerung auf kommunaler Ebene verbessert werden sollte [14].

Das AFOOT-Projektteam entwickelte auf Basis einer Dokumentenanalyse und von Workshops mit Akteur*innen Indikatoren für bewegungsfördernde Wohnumwelten. Diese wurden im Rahmen von Expert*inneninterviews mit Akteur*innen aus regionalen Institutionen im Bereich Planung, Verkehr und Gesundheit sowie mit Wissenschaftler*innen aus den Bereichen Stadtplanung, Verkehr, Gesundheit und mit Senior*innen rückgekoppelt. Die neu entwickelte Arbeitshilfe „Aktive Mobilität im Alter fördern“ [15] wurde von regionalen und überregionalen Multiplikator*innen evaluiert. Um auch die Perspektive von Bewohner*innen aufzunehmen, führte das AFOOT-Projektteam einen Survey mit älteren Menschen in der Metropolregion Nordwest durch, um aktive Mobilität auf Alltagswegen zu erfassen und fördernde oder hinderliche Faktoren der gebauten Umwelt und der Infrastruktur für das Zufußgehen und Fahrradfahren in Klein- und Mittelstädten zu identifizieren. Die Einrichtung eines Reallabors erfolgte in der Gemeinde Ritterhude (Landkreis Osterholz, Niedersachsen) mit der Zielsetzung, für aktive Mobilität und gesundes Altern in Kommunalverwaltung und -politik sowie in der Bevölkerung zu sensibilisieren. Die Zusammenarbeit zwischen Akteur*innen aus Gesundheitsförderung und räumlicher Planung sollte ebenso wie die Qualifizierung von baulichen und planerischen Maßnahmen hinsichtlich der Förderung von aktiver Mobilität gestärkt werden. Ergänzend dazu wurde eine Toolbox zur Konkretisierung und für die Implementation der einzelnen Bausteine prozessbegleitend entwickelt.

Dieser Diskussionsbeitrag baut auf den Ergebnissen des Projekts AFOOT auf und gibt einen Überblick zu raumbezogenen Zusammenhängen zwischen Gesundheit im Alter, Wohnumweltfaktoren und aktiver Mobilität (Zufußgehen, Radfahren) zum Erreichen von Alltagszielen. Wir diskutieren Ansätze für eine Verbesserung der räumlichen Gegebenheiten, Erfahrungen mit Methoden zur Beteiligung der älteren Bevölkerung in Planungsprozessen und Erkenntnisse für die Umsetzung von Maßnahmen.

## Aktive Mobilität – auch eine Frage der räumlichen Bedingungen

**Abb. 1:**
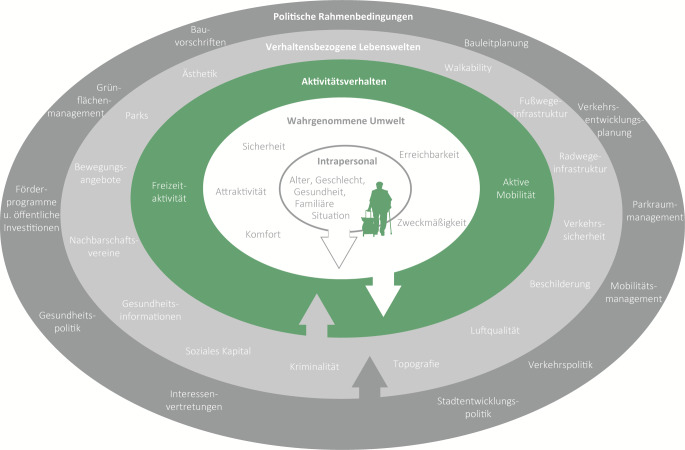
Einflussfaktoren auf aktive Mobilität und Freizeitaktivität nach dem Modell für ein aktives Leben, eigene vereinfachte Darstellung nach AFOOT-Projektteam [15], basierend auf Sallis et al. [16]

Die Mobilität von Menschen ist eine zentrale Voraussetzung für ihre soziale und wirtschaftliche Teilhabe. Heterogene Lebensstile der Bevölkerung haben auch die Mobilitätsmuster entsprechend verändert. Das Modell von Sallis et al. ([16]; Abb. 1) wurde als ein ökologisches Konzept zur Gestaltung aktiver Lebenswelten konzipiert und zeigt die vielfältigen Determinanten für eine aktive Mobilität, bei der die Menschen mit ihren spezifischen Dispositionen und Lebenslagen sowie ihrer jeweiligen Wahrnehmung der Lebensumwelt (kognitive Karte/„mental map“) im Mittelpunkt stehen. Das Aktivitätsverhalten wird demzufolge von verhaltensbezogenen Lebenswelten und diese wiederum von den politischen Rahmenbedingungen geprägt.

Im Alter gewinnen ästhetische und funktionale Ausstattungsmerkmale des Raums an Bedeutung, da motorische und visuelle Einschränkungen zunehmen. So ist eine als sicher wahrgenommene, saubere und ansprechende Umwelt für ältere Menschen wichtig. Zum einen spielt die räumliche Orientierung für die Sicherheit eine wichtige Rolle. Aber auch die Angst vor Stürzen steigt, wenn man sich im öffentlichen Raum mit Hindernissen wie Bordsteinkanten, Mülltonnen, auf dem Gehweg geparkten Fahrrädern oder Rollern bewegt. Demzufolge haben die räumlichen Gegebenheiten einen Einfluss auf das Verhalten, denn entscheidend für eine aktive Mobilität ist die Bewegungsfreundlichkeit des näheren Wohnumfelds (Walkability) für ältere Menschen. Da Anzahl und Länge der Wege mit dem Alter kontinuierlich abnehmen, steigt die Bedeutung des unmittelbaren Wohnumfeldes für körperliche Aktivität und Mobilität. Walkability ist durch mehrere Faktoren definiert, wie eine hohe Bevölkerungsdichte, gemischte Flächennutzung und vernetzte Straßenverbindungen. Ebenso entscheidend ist eine niedrigschwellige Erreichbarkeit von verschiedenen Dienstleistungen, Bushaltestellen, Parks und öffentlichen Räumen, um die Wege dorthin zu Fuß oder mit dem Fahrrad zurücklegen zu können [17]. Überdies sind die Qualität der Fußwege und Zugänge zum Wohngebäude und die Straßenbeleuchtung sowie Möglichkeiten zum Ausruhen (Sitzgelegenheiten) von Bedeutung. Somit wird die Gestaltung der verhaltensbezogenen Lebenswelt von den politischen Rahmenbedingungen verschiedener Politikfelder wie der Stadtentwicklungs‑, Verkehrs- und Gesundheitspolitik beeinflusst, die über Maßnahmen zur nachhaltigen Erhöhung der körperlichen Aktivität auf Bevölkerungsebene entscheiden.

Ein interdisziplinäres Handeln verschiedener Disziplinen, Akteur*innen und Entscheidungsträger*innen ist dabei die Voraussetzung. Die Gestaltung der räumlichen Umwelt ist eine Intervention, also ein Eingriff in die Verhältnisse, in denen die Menschen leben, und fokussiert auf alle Rahmenbedingungen, die deren Gesundheit beeinflussen. Dazu gehören die Qualität der Umwelt, wie beispielsweise die Außenluft, die Lern‑, Arbeits- und Wohnbedingungen, die soziale Lage und die Infrastruktur sowie das Angebot und der Zugang zu gesundheitlicher Versorgung und allgemeine politische Rahmenbedingungen. Diese stehen in einem wechselseitigen Bezug mit den individuellen Verhaltensweisen der Menschen.

Die absoluten Bevölkerungszahlen, die Zusammensetzung nach Alter, Geschlecht und Nationalität sowie weitere sozioökonomische und -demografische Charakteristika entwickeln sich regional unterschiedlich. Die demografische Entwicklung ist eine große Herausforderung für öffentliche Träger in der Ausgestaltung ihrer Aufgaben der Daseinsvorsorge. Disperse Wohnstandorte, vor allem im ländlich geprägten peripheren Raum sowie vielfach auch im Umland von Agglomerationsräumen, und auf zentrale Orte konzentrierte Versorgungs- und Dienstleistungsangebote erschweren die Teilhabe an Bildung, Gesundheitsversorgung, Kultur etc. und erfordern somit ein hohes Maß an Mobilität [18]. Diese kann jedoch insbesondere in ländlichen Räumen aufgrund größerer Entfernungen nur begrenzt aktiv erfolgen.

Gesundheitsfördernde Elemente der räumlichen Planung sind besonders wichtig für junge und ältere Menschen sowie für Personengruppen mit eingeschränkter Mobilität. Allerdings wird vonseiten der Stadtplanung zumeist die Meinung vertreten, dass die Zielsetzung gesunder Lebensverhältnisse implizit in den Handlungsfeldern, Strategien und Maßnahmen räumlicher Planung enthalten sei. Schaut man in Bezug auf die ältere Bevölkerung genauer hin, zeigt sich in dem Prozess des Alterns, dass ältere Menschen als heterogene Bevölkerungsgruppe unter sehr unterschiedlichen Bedingungen leben. Einkommen und körperliche Funktionsfähigkeit im früheren Lebensalter bestimmen die körperliche und geistige Gesundheit im höheren Lebensalter [19]. Soziale Benachteiligungen, zu denen auch die Wohnbedingungen, einschließlich der gebauten Wohnumwelt, gehören, sollten durch räumliche Planung ausgeglichen werden. Dafür werden Erkenntnisse über die Rahmenbedingungen und Perspektiven älterer Menschen auf die raumrelevanten Merkmale ihrer Wohnumgebung und ihrer Alltagsgestaltung benötigt.

## Aktive Mobilität und Perspektiven älterer Menschen auf die Qualitäten des öffentlichen Raums

Studien haben gezeigt, dass die Gestaltung der gebauten Umwelt die täglichen Aktivitäten außerhalb der Wohnung sowie das transportbezogene Zufußgehen und Fahrradfahren von älteren Menschen fördern kann [5, 17]. Die Forschung im Bereich der Einflussfaktoren der Wohnumwelt in Hinblick auf das Zufußgehen auf Alltagswegen beschränkt sich jedoch bisher auf größere Städte in Nordamerika oder Asien [17, 20]. Daten zu Klein- und Mittelstädten in Deutschland fehlen weitgehend. In dem Projekt AFOOT haben wir daher das aktive Mobilitätsverhalten Älterer in der Metropolregion Bremen-Oldenburg, die Bedeutung von Qualitäten des öffentlichen Raums und Präferenzen für die Gestaltung einer alternsgerechten Wohnumgebung untersucht. Ein besonderes Interesse galt den Einflussfaktoren auf Fahrrad- und E‑Bike/Pedelec-Fahren als Mobilitätsoption zum Erreichen von Alltagszielen im Alter, da die bisherige Forschung zu diesem Thema noch Lücken aufweist [17]. Das Fahren eines E‑Bikes/Pedelecs ist insbesondere für ältere Menschen mit gesundheitlichen Einschränkungen eine Möglichkeit, noch aktiv mobil zu sein, und es ermöglicht gerade im ländlichen Raum, trotz größerer Entfernungen Alltagsziele zu erreichen [21].

Im Jahr 2019 führten wir eine Querschnittstudie mit schriftlicher Befragung älterer Erwachsener durch. Es wurden zufällig ausgewählte 11.000 Erwachsene im Alter ab 65 Jahren kontaktiert, die in 11 Landkreisen und 2 Stadtgemeinden (< 100.000 Einwohner*innen) in der Metropolregion Nordwest in Deutschland lebten. Es nahmen 2242 Personen an der Befragung teil. Die Analysen zu aktiver Alltagsmobilität wurden in mehreren Artikeln publiziert [21–24].

77 % der befragten Frauen und 87 % der Männer hatten ein normales Fahrrad und/oder E‑Bike/Pedelec und/oder Dreirad. Einen Führerschein besaßen 88 % Frauen und 97 % Männer, 82 % Frauen und 93 % Männer verfügten jederzeit über einen Pkw. In Tab. 1 sind die Prävalenzen für Zufußgehen und Radfahren, um Alltagsziele zu erreichen, dargestellt [21, 22]. Die Prävalenz der aktiven Alltagsmobilität war geringer im hohen Alter, bei schlechter selbstberichteter Gesundheit, körperlicher Mobilitätseinschränkung, niedriger Bildung und niedrigem Einkommen. Die höchste Prävalenz des Zufußgehens war in Mittelstädten (20.000 bis <100.000 Einwohner*innen) zu beobachten. Demgegenüber waren die Prävalenzen des Radfahrens in Kleinstädten (5000 bis <20.000 Einwohner*innen) am höchsten.

**Tab. 1:** Prävalenz (%) der aktiven Alltagsmobilität älterer Menschen ab 65 Jahren zum Erreichen von Alltagszielen in der Metropolregion Nordwest

	Zufußgehen überhaupt	Zufußgehen mind. 3 ×/Woche	Radfahren (alle Radtypen) überhaupt	Radfahren (alle Radtypen) mind. 3 ×/Woche	E‑Bike/Pedelec-Fahren überhaupt	E‑Bike/Pedelec-Fahren mind. 3 ×/Woche
*Gesamt*	71,3	54,1	64,5	41,6	35,8	31,0
*Geschlecht*						
Frauen	70,8	51,2	59,3	38,2	30,9	24,7
Männer	71,4	55,9	68,1	43,6	39,6	35,9
*Alter*						
65–69	71,3	55,5	73,8	46,5	46,1	39,9
70–74	74,3	56,2	71,4	47,7	42,8	38,7
75–79	73,6	54,9	62,3	39,8	33,7	31,5
≥80	64,7	47,8	44,4	27,4	20,7	14,9
*Selbstberichtete Gesundheit*						
Gut/sehr gut	75,5	58,5	74,6	50,4	46,0	43,5
Mittelmäßig/schlecht/sehr schlecht	66,0	47,6	50,2	28,6	25,6	20,1
*Mobilitätseinschränkung*						
Keine	76,6	59,2	74,9	50,0	46,3	43,1
Mindestens eine	63,9	45,8	47,1	27,7	23,8	19,8
*Bildung*						
Hoch	76,0	59,4	69,5	43,0	35,9	29,7
Mittel	70,7	52,6	64,3	43,1	38,1	33,8
Niedrig	62,0	43,9	45,6	25,8	24,6	21,7
*Einkommen*						
Hoch	72,7	56,6	68,2	43,5	39,4	34,9
Mittel	72,9	53,9	62,1	40,8	32,8	28,0
Niedrig	68,2	45,2	58,0	37,8	33,3	29,8
*Wohnort*						
Mittelstadt	75,2	56,8	64,0	40,9	30,5	21,1
Größere Kleinstadt	71,4	53,3	64,4	43,7	37,8	37,0
Kleinere Kleinstadt	69,1	52,3	65,7	39,2	39,9	33,7
Landgemeinde	66,5	53,5	62,3	41,1	31,0	28,3

Zusammenfassende Ergebnisdarstellung, Details zu Erhebungsmethode, Kategorisierungen und Anzahlen sind den AFOOT-Publikationen [21, 22] zu entnehmen

**Abb. 2:**
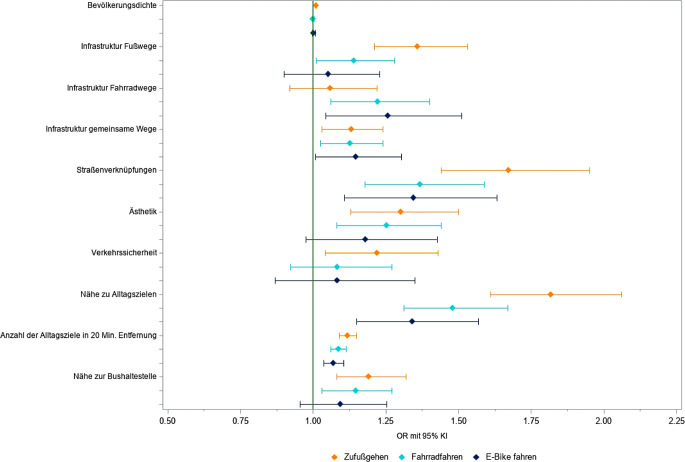
Assoziation zwischen Wohnumweltfaktoren und aktiver Alltagsmobilität älterer Menschen. Dargestellt sind adjustierte Odds Ratios (*OR*) mit 95 % Konfidenzintervall (*KI*). Details zu Methoden sind den AFOOT-Publikationen [21, 22] zu entnehmen. Quelle: eigene Abbildung

Mehrere Wohnumweltfaktoren waren mit aktiver Mobilität im Alter assoziiert [21, 22]. Dies betrifft vor allem die Nähe von Alltagszielen, ein dichtes Straßennetz/gute Wegeverbindungen und eine gute Fußwege- und Fahrradinfrastruktur. Die Assoziation des E‑Bike/Pedelec-Fahrens mit Wohnumweltfaktoren war etwas geringer im Vergleich zu Radfahren (alle Radtypen) oder Zufußgehen (Abb. 2).

In der befragten Population war ein E‑Bike/Pedelec relevant für das Erreichen von Alltagszielen, insbesondere in der Altersgruppe 65–74 Jahre sowie bei in Kleinstädten lebenden Personen. Die Anzahl gefahrener Minuten pro Woche mit einem E‑Bike/Pedelec lag bei Befragten mit Mobilitätseinschränkungen höher als bei denjenigen ohne [21]. Aktive E‑Mobilität bietet Potenziale für die Aufrechterhaltung einer eigenständigen aktiven Mobilität im Alter für die Gesundheit sowie für das Zurücklegen auch etwas weiterer Strecken [25].

Präferierte Gestaltungsmerkmale des Straßenraums umfassten verschiedene Aspekte der Sicherheit und der Aufenthaltsqualität im öffentlichen Raum, wie z. B. Sicherheit in Bezug auf Verkehrsunfälle oder Kriminalität, eine hohe Oberflächenqualität und eine gute Beleuchtung sowie als städtebauliche Gestaltqualität barrierefreie Wege und viel Platz zum Gehen [23]. Das Gefühl der Kriminalitätssicherheit wurde am häufigsten als wichtig eingestuft. Die höchsten Werte bei den Gestaltungspräferenzen wurden bei Frauen, Personen im Alter 80+, Menschen mit geringer Bildung und aktiv mobilen Menschen beobachtet. Mit steigender Gemeindegröße wurde eine zunehmende Präferenz für Beleuchtung, Kreuzungen und getrennte Rad- und Fußwege sichtbar. Die unterschiedliche Relevanz von städtebaulichen Gestaltungsmerkmalen für verschiedene Gruppen unterstreicht die Bedeutung einer vielfältigen Repräsentation in Beteiligungsprozessen der Städteplanung für eine alternsgerechte Stadtentwicklung. Stadtplanung muss diese unterschiedlichen Anforderungen kennen [23]. Ihre Aufgabe ist es im Kern, für potenzielle Konflikte bauliche, technische oder organisatorische Lösungen in der Abwägung der unterschiedlichen Interessenlagen zu entwickeln.

## Integrierte Indikatoren zur Erfassung von Mobilitätsanforderungen älterer Menschen

Für eine Verbesserung der räumlichen Gegebenheiten sind Stadt- und Verkehrsplanung in erster Linie gefragt. Um die Situation vor Ort zu erfassen, wurden im Projekt AFOOT integrierte Indikatoren mit objektiv messbaren und subjektiv erfassten Kennwerten definiert und den zentralen Themen, die sich auf die Bevölkerung und die gebaute Umwelt beziehen, zugeordnet. Diese sind bezogen auf die Individuen Soziodemografie, Wohlbefinden und Mobilitätsverhalten sowie bezogen auf den Raum öffentliche Räume, Rad- und Fußwegeinfrastruktur und Alltagsziele. Eine Betrachtung der gesundheitlichen Chancengerechtigkeit verknüpft die Bereiche miteinander. Details zu den Indikatoren sind in der AFOOT-Arbeitshilfe [15] zu finden.

Inwiefern angesichts der bereits großen Vielfalt an vorliegenden Daten noch weitere Datengrundlagen und Leitfäden für deren Erhebung und Einbeziehung in Planungsprozesse benötigt werden, wird aus stadtplanerischer Perspektive oftmals infrage gestellt, denn städtebaulichen Konzepten und Planungen liegt im Regelfall eine Analyse der (sozio)demografischen Ausgangslage zugrunde. Mit Blick auf Handlungsbedarf für eine ältere Bevölkerung sind jedoch Schrumpfungs- und Wachstumstendenzen möglichst kleinräumig auf Quartiersebene abzuschätzen und zu lokalisieren. Dazu gehören insbesondere Alter, Geschlecht, Bezug von Transferleistungen (Sozialhilfe) und Nationalität. Auch die Haushaltsgröße kann Hinweise auf Unterstützungsbedarf geben, bedenkt man den hohen Anteil der Einpersonenhaushalte älterer Menschen. Aufwändiger ist es, das Thema des Wohlbefindens zu erfassen, denn es bezieht sich auf den subjektiven Gesundheitszustand, einschließlich etwaiger Mobilitätseinschränkungen und der Nutzung von Gehhilfen. Dies kann nur durch eine Befragung erfasst werden. In erweiterten Analysen können Faktoren, wie beispielsweise Gestaltungspräferenzen, erfasst werden [26].

Das Mobilitätsverhalten älterer Menschen kann durch einen ortsbezogenen differenzierten Modal Split (prozentuale Verteilung des Verkehrsaufkommens (Wege) oder der Verkehrsleistung (Personenkilometer) differenziert nach verschiedenen Verkehrsmitteln) erfasst werden. Bei einem Fokus auf das Radfahren und Zufußgehen ist es empfehlenswert, die Anzahl der Wege mit einem Verkehrsmittel und nicht die mit dem Verkehrsmittel zurückgelegte Strecke zugrunde zu legen und die Nutzungshäufigkeit verschiedener Verkehrsmittel zu erfassen [26].

Die durch den Mixed-Methods-Ansatz in AFOOT (siehe Einleitung) gewonnenen Ergebnisse zeigen, dass für ältere Menschen die Gestaltung des öffentlichen Raums und seine Ausstattung von besonderer Bedeutung sind. Oftmals ist es wichtig zu wissen, ob es öffentliche Toiletten und Sitzgelegenheiten an den Wegen und Orten gibt. Für das Erreichen von Alltagszielen sind auch die Rad- und Fußwegeinfrastruktur entscheidend und somit nicht nur Geh- und Radwege in ihrer Ausgestaltung, sondern auch Straßenquerungen und Fahrradabstellanlagen. Für Kommunikation, Begegnung und Aufenthalt im Freien sollen multifunktionale Räume attraktiv für alle Generationen sein. Neben der Qualität der Infrastruktur ist ebenso die Nähe zu Alltagszielen, wie Supermärkten, Cafés, öffentlichen Treffpunkten und Grünanlagen wesentlich für die Möglichkeit, Zufußgehen und Fahrradfahren als Alltagsmobilität auch für die ältere Bevölkerung zu etablieren. Ein Überblick über die Versorgung mit diesen Alltagszielen und deren Erreichbarkeit in der eigenen Gemeinde ist in den Kartengrundlagen für einen Flächennutzungsplan bzw. für ein Stadtentwicklungskonzept gesamtstädtisch und auch teilräumlich zu erfassen. Denn es ist Aufgabe von Stadtplanung, Standorte für zu erhaltende bzw. fehlende soziale Versorgungsinfrastruktur zu identifizieren, um die nahräumliche Lebensqualität der Bevölkerung zu sichern (vgl. Baugesetzbuch § 1 Absatz 6, Satz 3). Hierbei bedarf es eines Blicks auf spezifische Ziele älterer Menschen, wie beispielsweise Parks und Friedhöfe, sowie Angaben zu Erholungsflächen (Gesamtfläche von Grünanlagen, Sportplätzen, Nutzflächen für Tiere und Pflanzen). Grünanlagen, Landwirtschafts‑, Wald- und Wasserflächen liegen im Regelfall auf Basis der amtlichen Flächenerhebung vor. Aus dem Vergleich von den Indikatoren zu den oben genannten 6 zentralen Themen und zum Querschnittthema gesundheitliche Chancengerechtigkeit zwischen Teilräumen und der Gesamtstadt lässt sich ableiten, ob Handlungsbedarf für eine verbesserte städtebauliche Gestaltung oder verkehrliche Organisation besteht.

Die Etablierung eines Monitoringsystems im Bereich von Mobilität ist empfehlenswert, um die Durchführung und Evaluierung von Maßnahmen zu unterstützen, frühzeitig auf problematische Entwicklungstrends aufmerksam zu werden und Prioritäten zu setzen. Dazu gehören das (kleinräumige) Monitoring der demografischen Entwicklung, insbesondere in Quartieren mit einem hohen Anteil von Kindern und Jugendlichen bzw. älteren Erwachsenen, und deren sichere Möglichkeiten für eine selbstständige Mobilität, unabhängig vom Auto. Ebenso könnten regelmäßig Online-Bevölkerungsbefragungen zu Gesundheit und Wohlbefinden oder Zukunftswerkstätten mit allen Generationen durchgeführt werden.

Zur Vermeidung umweltbezogener Ungleichheiten ist es für die Stadtplanung hilfreich, wenn Daten aus verschiedenen Verwaltungsbereichen zu Umweltressourcen (wie Grünflächenausstattung) und zum Vorkommen von Umweltbelastungen (wie Lärmbelastungen) im Vergleich zwischen Quartieren identifiziert und mit soziodemografischen Daten verknüpft werden können. Dabei kann oftmals auf vorhandene Daten in unterschiedlichen Ressorts in der Kommune oder in der Region zurückgegriffen werden [8].

## Methoden zur Beteiligung der älteren Bevölkerung

In vielen Teilen der Bevölkerung ist der Wunsch, an politischen Entscheidungsprozessen mitzuwirken, gewachsen und sie möchten ihre Perspektiven und Belange in Planungsprozesse einbringen. Dies wird oftmals als ein Mehraufwand für das Verwaltungshandeln gesehen, dient aber der besseren Information aller. Beteiligung führt nicht nur potenziell zu einer höheren Qualität von zielgerichteten Planungsmaßnahmen, sondern auch zu deren besseren Akzeptanz in der Bevölkerung. Sie dient mit der Zielsetzung der Gesundheitsförderung – im Sinne eines Empowerments – zugleich der Befähigung zu selbstbestimmtem Handeln und Einflussnahme auf die eigenen Lebensumstände. Gleichzeitig kann die Expertise in der Verwaltung von den Alltagserfahrungen profitieren. Zudem können Konfliktpotenziale frühzeitig erkannt und im Dialog zwischen Politik, Verwaltung und Bürgerschaft ausgehandelt werden. Mit Blick auf Chancengerechtigkeit ist auf die Einbeziehung von Minderheiten oder Personen mit nachteiliger sozioökonomischer Position besonderes Augenmerk zu legen. Dabei werden auch divergierende Interessenslagen offenbar, für die seitens der Stadtplanung technische und rechtliche Lösungen gefunden werden müssen.

In städtebaulichen Planungen in bestehenden (und benachteiligten) Quartieren sind Partizipationsprozesse inzwischen gesetzlich im „Besonderen Städtebaurecht“ im Baugesetzbuch vorgegeben. Um ältere Menschen anzusprechen, ist eine aufsuchende Beteiligung, bei der man dorthin geht, wo Bewohner*innen anzutreffen sind, geeignet, z. B. in Gemeindezentren, bei organisierten Treffen („Kaffeerunde“) oder in Vereinsheimen. Die Uhrzeit muss sorgfältig überlegt sein und die Orte sollten barrierefrei und gut erreichbar sein (zentral gelegen, mit guter Busanbindung). Mögliche partizipative Methoden sind [15]:

- Fokusgruppengespräche: Gruppendiskussion mit Bewohner*innen zu einem speziellen Thema;
- World Cafés: Verschiedene Themen werden an mehreren Tischen diskutiert. Nach einer bestimmten Zeit wechseln die Personen den Tisch;
- Spaziergangsgruppen: gemeinsamer Spaziergang mit Bewohner*innen durch die Nachbarschaft, um Probleme und Besonderheiten aufzuzeigen;
- Community Mapping: Bewohner*innen zeichnen eine Karte ihrer Nachbarschaft und tragen die für sie wichtigen Orte und Zielpunkte ein;
- Photovoice: Bewohner*innen bringen eigene Fotos von Problemstellen oder guten Beispielen mit, die gemeinsam diskutiert werden.

Eine Multiplikator*innen-gestützte Befragung, die durch geschulte Multiplikator*innen aus der entsprechenden Alters- bzw. Statusgruppe durchgeführt wird, setzt an der konkreten Lebenswelt an und dient der Wissensgenerierung und Aktivierung. Durch diesen potenziell besseren Zugang zu den Befragten können auch diejenigen angesprochen werden, die durch eine einfache postalische Übermittlung eines Fragebogens nicht erreicht würden. Dies findet gewöhnlich in einem Setting, d. h. einer Lebenswelt wie einer Seniorenbegegnungsstätte oder einer Nachbarschaft, statt und somit in einem vertrauten Kontext. Die Multiplikator*innen suchen die Bewohner*innen auf, sie erläutern den Sinn und die Bedeutung der Befragung und können ggf. beim Ausfüllen des Fragebogens unterstützen.

Bewährt haben sich ebenfalls Zukunftswerkstätten nach dem Konzept von Robert Jungk aus den 1960er-Jahren, um durch die Einbeziehung der Bürgerschaft in Prozesse der Entscheidungsfindung die Demokratie zu stärken [27]. Durch die strukturierte phasenweise Erarbeitung können die Teilnehmenden selbst die relevanten Aspekte definieren und werden zur Lösungsfindung und ggf. zur Mitwirkung an der Umsetzung der eigenen Ideen motiviert. Auch hier sind der Ort der Durchführung, eine angemessene Tageszeit sowie die Aufnahmefähigkeit und Mitwirkungsbereitschaft älterer Menschen zu bedenken. In der Stadtplanungspraxis ist die Umsetzung von solchen breit gefächerten Beteiligungsangeboten jedoch oftmals aufgrund mangelnder Kompetenzen und begrenzter zeitlicher und personeller Ressourcen eingeschränkt.

## Strategien und Maßnahmen zur Umsetzung

Mit dem Rückhalt von Kommunalpolitik und Verwaltungsspitze ist ein fachgrenzenüberschreitendes Verwaltungshandeln im Sinne des „Health-in-all-Policies“-Ansatzes und mit klar definierten Zuständigkeiten notwendig, z. B. für die räumliche Analyse oder die Umsetzung von Maßnahmen. Dabei ist zu klären, welche relevanten Akteur*innen aus den unterschiedlichen Sektoren hilfreich sind, z. B. bei der Bereitstellung von Datengrundlagen, aber auch ggf. als wichtige Kooperationspartner*innen für die Finanzierung von Maßnahmen. Ebenso sind themenbezogene Kooperationsstrukturen innerhalb der Verwaltung ein wichtiger Baustein. Bei diesen werden alle Mitarbeiter*innen der Verwaltung zusammengebracht, deren Arbeit das Themenfeld berührt, wie es z. B. beim Kompetenzteam „Gesundheit im Alter“ im Gesundheitsamt Köln der Fall ist [28]. Ein weiterer wichtiger Baustein ist das Einbringen des Themas in bestehende themenbezogene Gremien, z. B. Fachausschüsse. Des Weiteren können themenunabhängige Austauschplattformen innerhalb der Verwaltung genutzt werden (z. B. Treffen der Fachdienstleitungen, Vernetzung mit anderen Städten, Gemeinden und Landkreisen (z. B. Gesundheitsregionen Niedersachsen)) oder auch eine projektbezogene Zusammenarbeit initiiert werden (z. B. „Tag des guten Lebens“ [29]). Schließlich gilt es auch, bestehende Kooperationsstrukturen im Stadtteil oder in der Gemeinde zu nutzen. Diese Vorgehensweisen sind jedoch in kreisangehörigen Klein- und Mittelstädten schwieriger, vor allem aufgrund einer fachlich wenig ausdifferenzierten Verwaltungsstruktur und eines Öffentlichen Gesundheitsdienstes auf Kreisebene, der nicht vor Ort ansässig ist.

Leitlinien und Arbeitshilfen, die auf Basis interdisziplinärer gesundheitswissenschaftlicher und raumplanerischer Forschung und Expertise erarbeitet werden und von Stadtplaner*innen in ihrer täglichen Arbeit genutzt werden, stellen einen effektiven Wissenschafts-Praxis-Transfer dar [11]. Im Projekt AFOOT haben wir eine Arbeitshilfe entwickelt, die insbesondere Klein- und Mittelstädte dabei unterstützen soll, durch die Gestaltung einer bewegungsfördernden und alternsgerechten Umgebung aktive Mobilität in Form von Zufußgehen und Radfahren im Alter zu fördern. Sie ist in erster Linie für die Arbeit der kommunalen Planungs- und Bauverwaltung sowie des Öffentlichen Gesundheitsdienstes und deren intersektorales Verwaltungshandeln ausgelegt [15]. Die praxiserprobte Toolbox „Aktive Mobilität im Alter fördern“^2^ enthält eine Zusammenstellung praktischer Handreichungen für eine bewegungsfördernde und alternsgerechte Kommunalentwicklung. Sie enthält auch eine Sammlung konkreter Beispiele aus Kommunen zu den nachfolgend beschriebenen Handlungsstrategien.

In der AFOOT-Arbeitshilfe [15] werden 3 Handlungsstrategien formuliert, die auf die Umsetzung von Maßnahmen abzielen.

### Handlungsstrategie A: (Halb‑)öffentliche Räume multifunktional gestalten und ausstatten

Für die zwischenmenschliche Kommunikation, das Aufeinandertreffen gleichgesinnter oder auch unterschiedlicher Bevölkerungsgruppen und als Orte der Begegnung mit Bekannten und Unbekannten spielen öffentliche Plätze, Parks und Straßen sowie halböffentliche Räume, wie Schulhöfe, Caféterrassen oder Gemeinschaftsgärten, eine zentrale Rolle im Alltagsleben der Bewohner*innen. Ihre Funktionsfähigkeit ist geprägt von den angrenzenden Nutzungen, z. B. Gastronomie, Einzelhandel und öffentliche Einrichtungen. Bei multifunktionaler Gestaltung können sie differenzierte Aufenthaltsangebote für unterschiedliche Nutzergruppen und Bedürfnisse eröffnen und zudem Angebote temporärer Nutzungen und Aneignungen bieten. Die Gestaltung öffentlicher Räume als Treffpunkte in einer Nachbarschaft (Third Places) ist für ältere Menschen, die ihre Kontakt- und Kommunikationsbedürfnisse nicht mehr in ihrem beruflichen Umfeld (Second Place) befriedigen können, von hoher Bedeutung. Mit Grün und ggf. auch Blau (Wasser) gestaltete Freiräume, witterungsgeschützte Aufenthaltsbereiche mit öffentlichen Toiletten und Trinkwasserangeboten, Sicherheit und Sauberkeit und eine gute Erreichbarkeit dieser Räume sind von zentraler Bedeutung. Zugleich bergen sie das Potenzial für eine übergeordnete Freiraumvernetzung. Vielseitig nutzbare (halb-)öffentliche Räume unterstützen das Gehen und Radfahren als Mittel zur Fortbewegung und erleichtern es insbesondere älteren Menschen, familiäre, freundschaftliche und nachbarschaftliche Kontakte aufrechtzuerhalten. All dies sind Voraussetzungen für eine autonome, gesunde und aktiv gestaltete Lebensqualität in jedem Alter [15].

### Handlungsstrategie B: Aktive Mobilität auf Alltagswegen priorisieren

Der große Vorteil der Fortbewegung aus eigener Kraft – ob zu Fuß, mit dem Zwei- oder Dreirad, mit dem Rollstuhl, Rollator, Roller oder Rollschuh – liegt darin, dass diese grundsätzlich zeitlich flexibel und für geringe Kosten verfügbar ist. Allerdings muss die räumliche Umgebung entsprechende Bedingungen bereithalten. Innerorts sind Geschwindigkeitsreduzierung, Querungsmöglichkeiten für den Fuß- und Radverkehr und Aufenthaltsqualität zu adressierende Aufgaben, während außerorts eine Verbindung einzelner Ortsteile und eine Vernetzung von Stadt und Umland wichtig sind. Hier gilt es, die Perspektiven und Belange von Zufußgehenden und Radfahrenden bei der Planung einzubeziehen, wie dies in den integrierten Verkehrsentwicklungskonzepten zwar bereits erfolgt, aber noch einer stärkeren Umsetzung bedarf. Denn Straßenräume müssen vor allem ausreichend Platz für weniger mobile Menschen bieten, ohne physische Barrieren und übersichtlich gestaltet sein, um im Alterungsprozess auftretende Einschränkungen z. B. von Kraft, Beweglichkeit und Balance sowie Hören und Sehen und abnehmende Reaktionsschnelle hinsichtlich der Verkehrssicherheit zu kompensieren. Zufußgehenden und Radfahrenden ist Vorrang zu gewähren mit barrierefreien, sicheren und ausreichend breit gestalteten Fuß- und Radwegen; übersichtliche und selbsterklärende Straßenräume sind zu schaffen. Insgesamt sind die Verkehrssicherheit und das subjektive Sicherheitsempfinden zu erhöhen und Rad- und Fußverkehr als Ergänzung des ÖPNV zu begreifen [15].

### Handlungsstrategie C: Erreichbarkeit von Alltagszielen durch Siedlungsentwicklung gewährleisten

Eine kompakte Siedlungsstruktur ermöglicht kurze Wege und eine Mischung von Wohnen, Einzelhandel, Dienstleistungen und sozialer Infrastruktur. Langfristige Infrastrukturkosten für die Unterhaltung neuer Siedlungsflächen müssen realistisch kalkuliert werden, z. B. mit dem Folgekostenrechner für Baugebiete. Dies erfordert in urban geprägten Räumen eine kommunale und regionale Entwicklungsplanung, die entsprechend den gesetzlichen Vorgaben auf Innenentwicklung fokussiert, um Fußgänger- und Radfahrfreundlichkeit zu erreichen. Eine altersbedingte Orientierung auf den Nahraum eröffnet die Chance, insbesondere für kleine Kommunen, dass altersgerechte Wohnungen dem Funktionsverlust der Ortskerne entgegenwirken und aktive Ältere das öffentliche Leben in der Kommune, z. B. durch ehrenamtliche Tätigkeiten, bereichern. Um wohnortnahe Versorgungsstrukturen aufrechtzuerhalten und zu fördern, können die Kooperation mit benachbarten Kommunen und die Organisation von (mobilen) Angeboten der Versorgung und Mobilität hilfreich sein [15].

In Bezug auf Handlungsstrategien und deren Umsetzung konnten wir in dem Projekt AFOOT zeigen, dass eine systematische Zusammenarbeit des Planungs- und Gesundheitssektors – hier in der Metropolregion Bremen-Oldenburg – bislang fehlt, insbesondere im Bereich der Bewegungsförderung im Alter durch Stadtgestaltung [30]. Durch die Integration des Themas der aktiven Mobilität in die tägliche Arbeit der Planungsverwaltung wurden die Rolle und Möglichkeiten der räumlichen Planung bei der Verbesserung der Gesundheit und des Wohlbefindens der Bevölkerung erkannt. Dies gilt auch in Bezug auf den Bedarf an lokalen Strategien in der Verwaltung, die das Zufußgehen, Fahrradfahren und den öffentlichen Nahverkehr priorisieren. Soziale Ungleichheiten in der Gesundheit zu reduzieren, spielte in der kommunalen Diskussion eine nachrangige Rolle.

Für die Gemeinde Ritterhude stellte eine Zusammenarbeit mit Akteur*innen der Gesundheitsförderung im Rahmen des Reallabors in dieser Form Neuland dar. Bislang gab es weder eine institutionelle Verankerung für das Thema Gesundheit noch eine systematische Zusammenarbeit mit dem zuständigen Gesundheitsamt des Landkreises. Auch eine systematische Erhebung oder Planung zur Förderung aktiver Mobilität im Alter gab es zuvor nicht. Das Reallabor sollte einen Rahmen schaffen, um Ansätze zur intersektoralen Zusammenarbeit zwischen Akteur*innen der Gesundheitsförderung und der räumlichen Planung und zur Förderung von aktiver Mobilität und gesundem Altern in der Praxis zu erproben. In den Zeitraum des Reallabors in Ritterhude fielen die Erarbeitung eines kommunalen Radförderkonzeptes und die Auseinandersetzung mit den raumbezogenen Bedürfnissen älterer Menschen im Seniorenbeirat sowie im Rahmen der Spielleitplanung (strategisches Instrument für eine kinder- und jugendgerechte Planung). Es gelang, in diese Routineverfahren des Verwaltungshandelns erstmals eine Gesundheitsförderungsperspektive einzubringen.

Bezüglich der räumlichen Rahmenbedingungen für aktive Mobilität und gesundes Altern stellten sich die Handlungsmöglichkeiten – jenseits der Interventionen zur Sensibilisierung – als gering heraus. Eine kurzfristige Geschwindigkeitsreduzierung auf Tempo 30 in der Hauptdurchgangsstraße von Ritterhude selbst nur für einen Aktionstag wurde vom Landkreis abgelehnt. Es wurde jedoch eine Begründung für einen Antrag zur dauerhaften Geschwindigkeitsreduzierung in dieser Straße auf Tempo 30 formuliert und in den Gemeinderat eingebracht. Des Weiteren wurde eine lokale Agenda „Aktiv mobil – länger gesund“ entwickelt. Die Größe der Gemeinde und ihre Verwaltungsstruktur sowie die kurze Laufzeit des Reallabors können als eher hemmende Faktoren für eine nachhaltige Verstetigung der Strategien und Maßnahmenumsetzung vermutet werden.

## Fazit zum Projekt AFOOT

Die Bedeutung einer aktiven Mobilität älterer Menschen mit dem Fahrrad oder zu Fuß, die gesundheitserhaltend und -fördernd wirken kann, und die Verhältnisse der räumlichen Strukturen und baulichen Gestaltung waren das zentrale Thema in dem Forschungsprojekt AFOOT. Wir entwickelten interdisziplinär Strategien und Maßnahmen zur Förderung aktiver Mobilität im Alter und erprobten diese initial in einem Reallabor. AFOOT-Ergebnisse belegen die Heterogenität der Bevölkerungsgruppe älterer Menschen in Bezug auf aktive Mobilität und deren Einflussfaktoren. Zugleich gelang es, sie für die räumliche Gestaltung ihrer Umwelt zu interessieren. Für eine aktive Mobilität bis in das hohe Alter sind sowohl räumliche Verhältnisse als auch deren Wahrnehmung von Bedeutung. Insbesondere in kleinen Gemeinden wurden aktive Mobilität und gesundes Altern als Themenfeld als eher wenig anschlussfähig wahrgenommen. Deutlich mehr Bezüge konnten die Vertreter*innen aus kreisfreien Städten herstellen. Landkreisvertreter*innen knüpften vor allem über die besonderen Bedürfnisse älterer Menschen an das Thema an. Die sektorübergreifende Zusammenarbeit von Stadtplanung, Verkehrsplanung und Public Health für die Förderung der aktiven Mobilität und der Gesundheit älterer Menschen ist zukünftig weiter zu stärken.

## Footnotes

1 AFOOT (Alternd zu Fuß oder mit Fahrrad: urban mobil ohne Stress): siehe https://www.uni-bremen.de/afoot; zugegriffen am: 28.05.2024.

2 Siehe: https://www.uni-bremen.de/afoot/toolbox; zugegriffen am: 28.05.2024.
